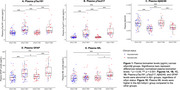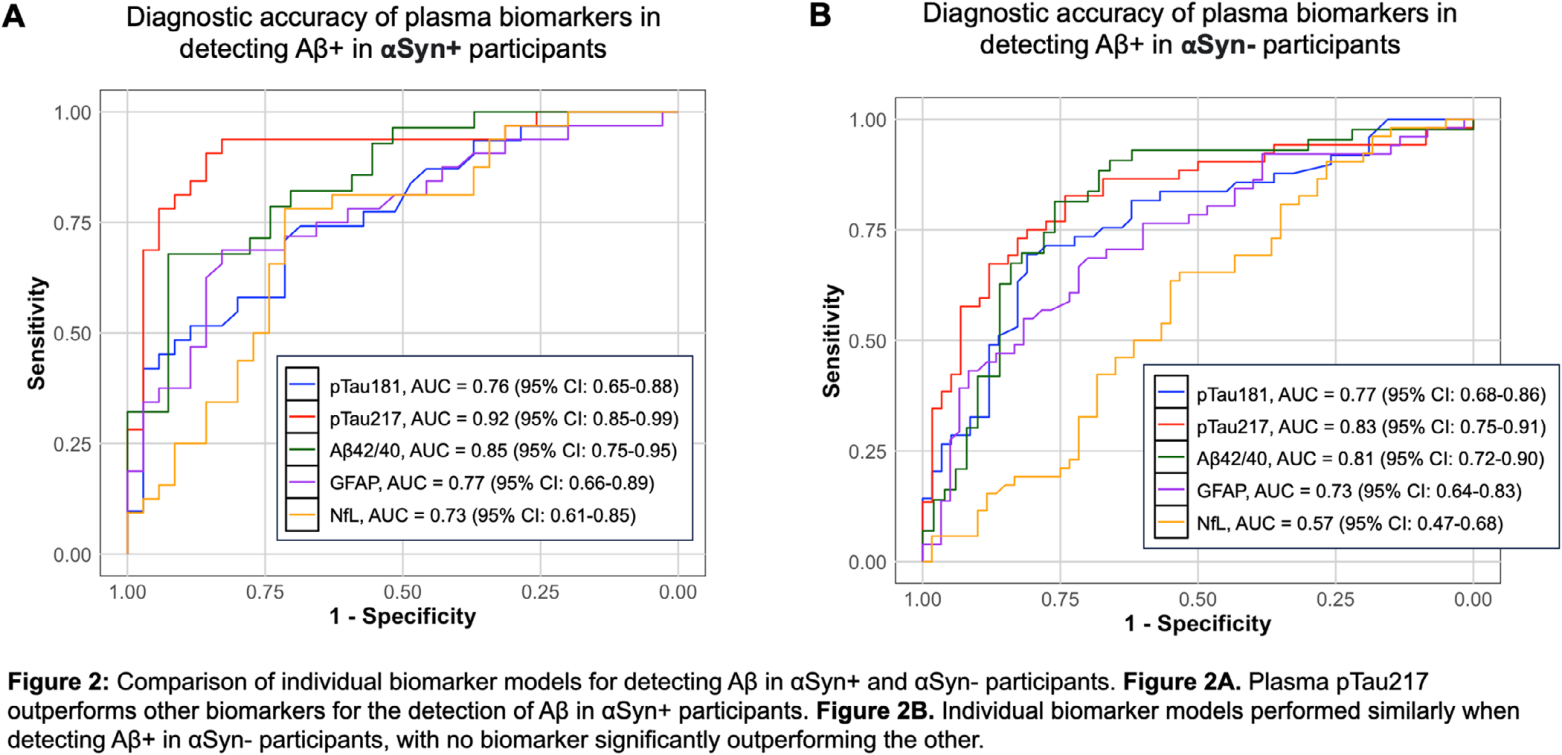# Diagnostic performance of plasma biomarkers for detecting amyloid co‐pathology in Neuronal α‐Synuclein Disease

**DOI:** 10.1002/alz70856_104596

**Published:** 2025-12-26

**Authors:** Alena Marie Smith, Sara A Lorkiewicz, Laia Montoliu‐Gaya, Ted N. Wilson, Nicholas Ashton, Burak Arslan, Melanie J. Plastini, Christina B. Young, Joseph R. Winer, Marian Shahid, Hillary Vossler, Veronica Ramirez, Tianyu Pan, Geoffrey A. Kerchner, Katrin I. Andreasson, Victor W. Henderson, Thomas J. Montine, Lu Tian, Elizabeth C. Mormino, Henrik Zetterberg, Kathleen L. Poston, Carla Abdelnour

**Affiliations:** ^1^ Stanford University School of Medicine, Stanford, CA, USA; ^2^ Department of Psychiatry and Neurochemistry, Institute of Neuroscience and Physiology, The Sahlgrenska Academy, University of Gothenburg, Mölndal, Sweden; ^3^ Department of Old Age Psychiatry, Institute of Psychiatry, Psychology & Neuroscience, King's College London, London, United Kingdom; ^4^ Department of Psychiatry and Neurochemistry, Institute of Neuroscience and Physiology, The Sahlgrenska Academy, University of Gothenburg, Mölndal, Gothenburg, Sweden; ^5^ Stanford University, Department of Statistics, School of Humanities and Sciences, Stanford, CA, USA; ^6^ F. Hoffmann‐La Roche Ltd, Basel, Basel, Switzerland; ^7^ Department of Neurodegenerative Disease, UCL Institute of Neurology, London, United Kingdom; ^8^ Department of Psychiatry and Neurochemistry, Institute of Neuroscience and Physiology, The Sahlgrenska Academy at the University of Gothenburg, Mölndal, Västra Götalands län, Sweden; ^9^ UK Dementia Research Institute, University College London, London, United Kingdom; ^10^ Clinical Neurochemistry Laboratory, Sahlgrenska University Hospital, Gothenburg, Sweden

## Abstract

**Background:**

Coexistence of amyloidosis (Aβ), tauopathy, and alpha‐synucleinopathy (αSyn) is common in neurodegenerative disease and drives heterogeneity in clinical presentation, disease progression, and treatment response. Understanding how in vivo biomarkers perform within the context of multiple underlying neuropathologies is therefore critical. While novel plasma biomarkers accurately detect Aβ, their application in the presence of αSyn remains underexplored. This study investigated the diagnostic accuracy of plasma biomarkers for detecting Aβ in individuals with and without αSyn pathology.

**Method:**

Five plasma biomarkers were analyzed (pTau181, pTau217, Aβ42/40, GFAP, and NfL) in a cohort of 180 Stanford research participants (mean age = 69); 48% were asymptomatic (healthy controls) and 52% were symptomatic (clinically diagnosed along the Alzheimer's or Lewy body disease spectra). The CSF SAAmplify‐αSYN test and Aβ42/40 ratio (Lumipulse G platform) were used to determine αSyn status (αSyn+ and αSyn‐) and Aβ status (Aβ+ and Aβ‐). Descriptive comparisons were conducted for plasma biomarkers across Aβ/aSyn groups (αSyn‐/Aβ‐, *n* = 60; αSyn‐/Aβ+, *n* = 53; αSyn+/Aβ‐, *n* = 35; and αSyn+/Aβ+, *n* = 32). Diagnostic accuracies of plasma biomarkers in predicting Aβ status were evaluated in αSyn+ and αSyn‐ individuals with receiver‐operating characteristic (ROC) curve analyses and compared with the DeLong test.

**Result:**

Plasma pTau181, pTau217, Aβ42/40, and GFAP levels were abnormal in Aβ+ groups, regardless of αSyn status (Figure 1). Plasma NfL levels were higher in the αSyn+/Aβ+ group compared to other groups. Plasma pTau217 showed the largest median fold change between Aβ+ and Aβ‐ participants. In αSyn+ participants, plasma pTau217 and Aβ42/40 individually showed the highest accuracies (AUC values up to 0.92) in detecting Aβ (Figure 2). In αSyn‐ participants, individual and combined biomarker models performed similarly, with no biomarker significantly outperforming another. Including age, sex, and APOE4 status did not improve model accuracy for detecting Aβ in αSyn+ participants, but improved plasma NfL's accuracy for detecting Aβ in aSyn‐ participants.

**Conclusion:**

Plasma pTau217 (individually and combined with other biomarkers) accurately detects Aβ in αSyn+ participants. These findings highlight potential for using plasma pTau217 as a tool for Aβ screening and stratification in clinical trials for alpha‐synucleinopathies such as dementia with Lewy bodies and Parkinson's disease.